# Hyphema: A Rare Complication of Periprocedural Antiplatelets and Anticoagulants Following Percutaneous Coronary Intervention

**DOI:** 10.7759/cureus.18609

**Published:** 2021-10-08

**Authors:** Amit Gulati, Rajneesh Calton, Cinosh Mathew, Sakshi Khurana, Nivedita Calton

**Affiliations:** 1 Internal Medicine, Maimonides Medical Center, Brooklyn, USA; 2 Cardiology, Christian Medical College and Hospital, Ludhiana, IND; 3 Cardiology, Smt. B. K. Shah (SBKS) Medical Institute and Research Center, Vadodara, IND; 4 Radiology, New York Presbyterian-Columbia University Irving Medical Center, New York City, USA; 5 Internal Medicine, Christian Medical College and Hospital, Ludhiana, IND

**Keywords:** hemorrhagic, anticoagulant, antiplatelets, myocardial reperfusion, hyphema

## Abstract

Hemorrhagic complications are one of the major complications encountered with reperfusion therapies. However, ocular hemorrhage, especially hyphema, i.e., bleeding into the anterior chamber of the eye is one of the rarest bleeding manifestations. Bleeding manifestations in the periprocedural period can be devastating for the patient as antiplatelets and anticoagulants may need to be stopped and this can lead to stent thrombosis. We present a case of a 55-year-old lady, who was a known diabetic and hypertensive and developed hyphema with periprocedural antiplatelets and anticoagulants following percutaneous coronary intervention (PCI). She was managed medically and the dose of antiplatelets was reduced. She was discharged once there was evidence of a reduction in hyphema. Two weeks post-discharge her hyphema had completely resolved.

## Introduction

The optimal treatment of a patient presenting with acute myocardial infarction (AMI) is to achieve coronary perfusion in the infarct-related artery as soon as possible. This has been given a class I indication in the American Heart Association (AHA) guidelines [1]. This can be achieved by percutaneous coronary intervention (PCI) or thrombolytic therapy, PCI being superior to thrombolysis in the majority of the cases [2]. However, bleeding is one of the major complications encountered in both reperfusion options. Hyphema or the presence of blood in the anterior chamber of the eye is one of the rarest bleeding manifestations for post PCI patients. Bleeding in the post PCI period can be catastrophic as sometimes, antiplatelets and anticoagulants may need to be stopped, which increases the chances of stent thrombosis.

We present a case of a 55-year-old lady, a known diabetic and hypertensive, who had developed hyphema with periprocedural antiplatelets and anticoagulants following PCI.

## Case presentation

A 55-year-old lady, a known diabetic and hypertensive for the last 10 years presented with symptoms of chest pain at rest for five days. Her blood pressure at the time of admission was 130/76 mmHg with a heart rate of 76 per minute. Her electrocardiogram (EKG) revealed ST depression in leads V1-V4, while the echocardiogram was normal. She had raised troponin levels and was diagnosed to have a non-ST-elevation myocardial infarction (NSTEMI). She underwent posterior chamber intraocular lens implantation in the left eye three years back. She followed an ophthalmologist outpatient and had no evidence of intraocular bleed in the past. After medical stabilization, she underwent coronary angiography (CAG), which revealed single vessel disease with 90% stenosis in the mid-left anterior descending (LAD) artery with a thrombus. After discussing treatment options with relatives she was taken up for PCI to LAD artery. She underwent uneventful PCI with stenting to LAD artery with Xience Prime 2.75 x 38 mm (Abbott Vascular, CA) stent. She was loaded with aspirin 325 mg and clopidogrel 300 mg prior to the procedure. During the procedure, she received 100 IU/kg of heparin along with tirofiban (GPIIb/IIIa inhibitor [GPI]) dose as per body weight. Activated clotting time (ACT) was maintained at 200-250 seconds during the procedure.

**Figure 1:**
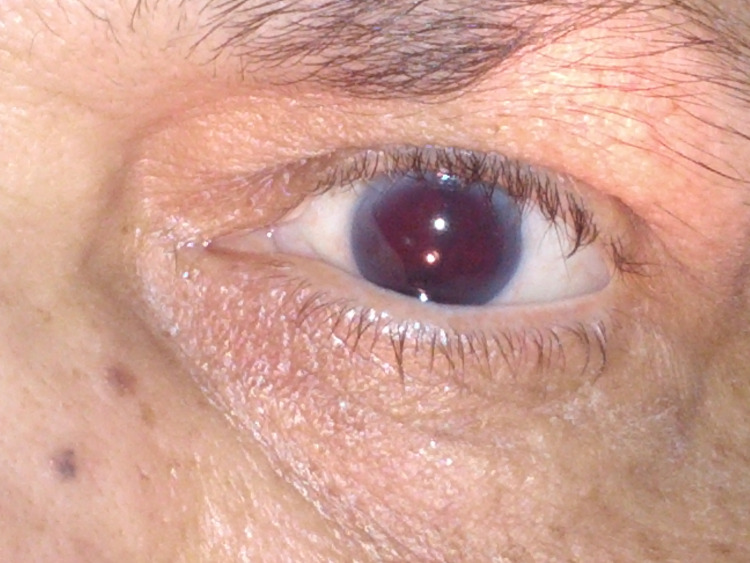
Post PCI, the patient had blurring of vision. Left eye examination under the torch light shows evidence of anterior chamber bleed.

Post procedure, she was shifted to the Intensive Cardiac Critical Care Unit on tirofiban infusion as she had a thrombus in the LAD artery and was asymptomatic. However, after one hour post PCI patient, she suddenly complained of blurring of vision and inability to see from the left eye. She was normotensive at that time with a BP of 128/82 mmHg. She was examined immediately and torch light examination revealed a reddish hue in her left eye (Figure 1).

An ophthalmic consult was immediately sought and she was diagnosed to have an anterior chamber bleed. The tirofiban infusion was immediately discontinued and ACT was checked which was 188 seconds at that time. She was given an upright position and was started on topical beta-blockers to decrease IOP. By evening, she started having a perception of light in the left eye, and by the next day morning, she could do finger counting at two feet. A slit-lamp examination was done, which confirmed hyphema without any raised intraocular pressure (IOP) changes. She was kept on 75 mg aspirin and 75 mg clopidogrel with regular aPTT monitoring and she improved over the next two days. Repeat examinations had shown that her hyphema had considerably reduced. She remained asymptomatic from a cardiac point of view and serial cardiac enzymes were normal and EKG did not show any new changes. She was discharged on dual antiplatelet and cardiac medications with timolol eye drops. On follow-up after two weeks, her hyphema had completely resolved and her IOP was normal.

## Discussion

In the GUSTO-I trial, among all the hemorrhagic complications in AMI treatment, ocular hemorrhage after reperfusion therapy was uncommon. Hyphema is defined as the presence of blood within the aqueous fluid of the anterior chamber [3]. It is most commonly caused by injury (blunt trauma and lacerating trauma). Hyphemas may occur spontaneously without an inciting cause due to neovascularisation, tumors of the eye like retinoblastoma, iris melanoma, or vascular anomalies like juvenile xanthogranuloma. Other medical causes of spontaneous hyphema include myotonic dystrophy, leukemia, and Von Willibrand disease. Antiplatelets, anticoagulants, and thrombolytic agents may cause hyphema by causing excessive thinning of the blood [3].

Patients undergoing PCI with stenting require dual antiplatelets for at least one year. Also, patients presenting with ACS with a high thrombus burden require intravenous antiplatelets in the form of GPIs along with anticoagulants post stenting. These measures are taken to prevent stent thrombosis. Patients who develop major bleeding manifestations post PCI are at an elevated risk of stent thrombosis as their antiplatelets need to be withheld. The most common site of bleed is from the access site followed by the oral cavity, GI tract, and urinary tract. The present case highlights the possibility of intraocular bleed which can be a rare site for bleeding post PCI. In this case, we had given the patient standard loading doses of antiplatelets and weight-adjusted heparin and GPIs and her ACT was in the therapeutic range. However, she developed an anterior chamber bleed, which is a rare site of bleed. This was a challenging situation as she needed to be on antiplatelets to avoid stent thrombosis. We managed the case by stopping GPI infusion and continuing her on dual oral antiplatelet medications in order to prevent stent thrombosis.

On reviewing the literature for the development of hyphema post PCI, no case showed such a complication. To the best of our knowledge, ours is the first case report of this kind.

We did find three case reports of retro-orbital hemorrhage occurring post PCI. Cunneen et al. presented a case report of a patient with retro-orbital bleeding which occurred after thrombolysis followed by coronary angioplasty for AMI [4]. Diatchuk et al. reported a 60-year-old man with spontaneous subperiosteal orbital hemorrhage following treatment with streptokinase and heparin for AMI [5]. Hsu-Ping Wu reported a similar case of retro-orbital hemorrhage [6].

Our patient, improved with reducing the dose of antiplatelets and stopping GPIs. The ophthalmologist started the patient on topical timolol. On follow-up, she had a complete recovery of her vision.

We, therefore, stress upon the fact that in patients with coronary artery disease who receive antiplatelet and antithrombotic agents, the development of anterior chamber bleed could be a rare and challenging situation. It requires prompt ophthalmological consultation and treatment as it can result in catastrophic vision sequel. When hemorrhage is present, the cardioprotective benefits of antithrombotic agents need to be weighed against the overall threat to the patient’s vision.

## Conclusions

Our case emphasizes that though the most common cause of hyphema is a traumatic injury to the globe, it can be a rare complication of antiplatelet and anticoagulation therapy. It does not typically cause permanent loss of vision, but its presence signifies considerable insult to the globe and therefore requires careful follow-up and management. Patient education is essential to minimize complications in the first several days after the injury as well as for the long-term ocular health of the patient.
